# Optic nerve haemangioblastoma in association with von Hippel-Lindau syndrome: case report and literature review

**DOI:** 10.1093/bjrcr/uaae007

**Published:** 2024-02-12

**Authors:** Juan David Vásquez Montoya, Jorge Mario Velez, Melisa Naranjo Vanegas, Natalia Montes Jimenez

**Affiliations:** School of Medicine, Universidad CES, Medellín 085006, Colombia; Medical imagine & AI in health SURA, Bioscience Center, Ayudas Diagnósticas SURA, Medellín 050015, Colombia; Medical imagine & AI in health SURA, Bioscience Center, Ayudas Diagnósticas SURA, Medellín 050015, Colombia; Medical imagine & AI in health SURA, Bioscience Center, Ayudas Diagnósticas SURA, Medellín 050015, Colombia

**Keywords:** haemangioblastoma, optic nerve, von Hippel-Lindau syndrome, case reports

## Abstract

Optic nerve haemangioblastoma (ONH) is an uncommon, benign, non-meningothelial, mesenchymal tumour of unclear origin. Most are associated with von Hippel-Lindau (VHL) syndrome (71%), and only 40 cases have been reported in the medical literature. Most of the patients develop non-specific visual symptoms, including decreased visual acuity and/or loss of visual fields, exophthalmos, trigeminal neuralgia, and retroorbital pain. Optic nerve sheath meningioma and optic nerve glioma are among the differential diagnoses that may be considered in this location. Contrast-enhanced MRI is considered an optimal diagnostic tool, which helps to determine some characteristics that guide towards an adequate diagnosis and treatment. We present a 42-year-old patient with a history of VHL syndrome in whom a cerebellar lesion and optic nerve lesions were evidenced, and we did a review of the literature and case analysis.

## Introduction

Optic nerve haemangioblastoma (ONH) is an infrequent and benign tumour, typically unilateral, and most in association with von Hippel-Lindau (VHL) syndrome. Haemangioblastomas typically constitute approximately 2% of the total number of tumours affecting the central nervous system (CNS), with a prevalence of 7%-12% specifically among tumours located in the posterior fossa. The tumour is categorized as a grade I non-meningothelial mesenchymal tumour of unknown aetiology according to the World Health Organization classification. The usual localization sites are in the brain (90%), of which 95% are in the cerebellum and 10% in the spinal cord. Other places are very rare, especially those involving the optic nerves and the chiasm.^1^

Optic nerve haemangioblastomas can occur sporadically or in association with VHL (71%). VHL disease is autosomal dominant that results in the inactivation of a tumour suppressor gene located on chromosome 3p25-26 and is diagnosed in early adulthood and causes multiple tumours, such as retinal haemangioblastomas (RH), CNS haemangioblastomas, endolymphatic sac tumours, renal cell carcinoma, and pheochromocytomas.^1^^,^^2^

On MRI, CNS haemangioblastoma (mostly in the cerebellum) is divided into 4 types based on its structure: cystic with mural nodule (54%), solid (28%), cystic (12%), and solid with cystic component (3.8%). The cystic component of the tumour is hypointense in the T1-weighted images and hyperintense in the T2-weighted images, and the solid component shows an avid enhancement in contrast-enhanced T1-weighted MR images. It has a flow void and does not restrict diffusion-weighted imaging (DWI). ONH presents some important characteristics that differentiate it from cerebellar haemangioblastoma, such as the smaller amount of a cystic component and moderate-to-severe peritumoural oedema. It is also different from other tumour lesions that have flow void and hyperintensity in the ADC map.^1^^,^^3^

## Case report

**Figure 1.**
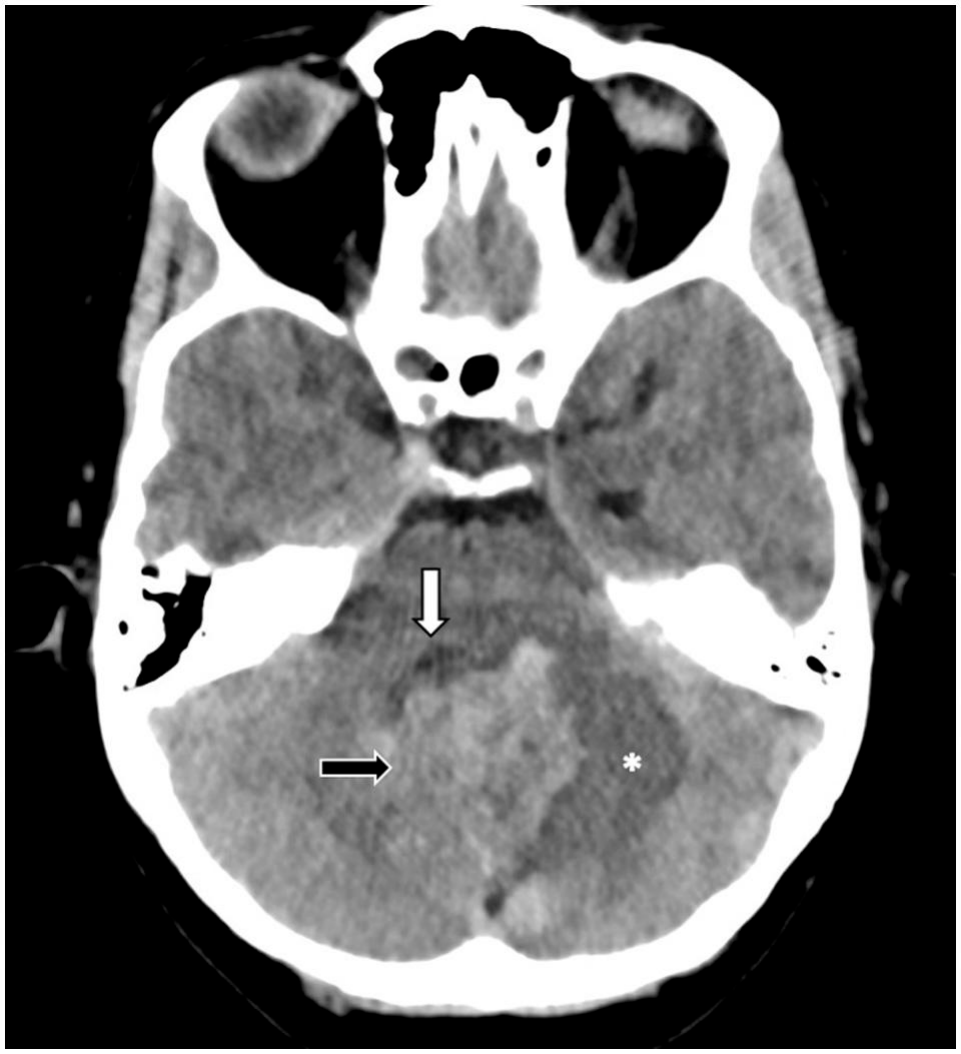
Non-contrast head CT shows a heterogeneous and hyperdense multilobulated mass (black arrow) in the left cerebellar hemisphere, with perilesional edema (asterisk) and compression on the fourth ventricle (white black).

A 42-year-old woman with a 6-month history of headaches irradiated to cervical region and with burning feeling, local numbness, dizziness, vomiting, worsening symptoms, and gait disorder. As part of her medical history, she had adrenal insufficiency 20 years ago, associated with surgical removal of bilateral pheochromocytoma and family history of brain tumor with unknown diagnosis in her father. During physical examination, patient’s blood pressure was high and there were signs of endocranial hypertension, obstructive hydrocephalus, and posterior ventriculoperitoneal shunt. Head CT Figure 1 showed a heterogeneous and hyperdense multilobulated mass in the left cerebellar hemisphere, with perilesional oedema and compression on the fourth ventricle infiltrative and brain stem and secondary obstructive hydrocephalus.

**Figure 2.**
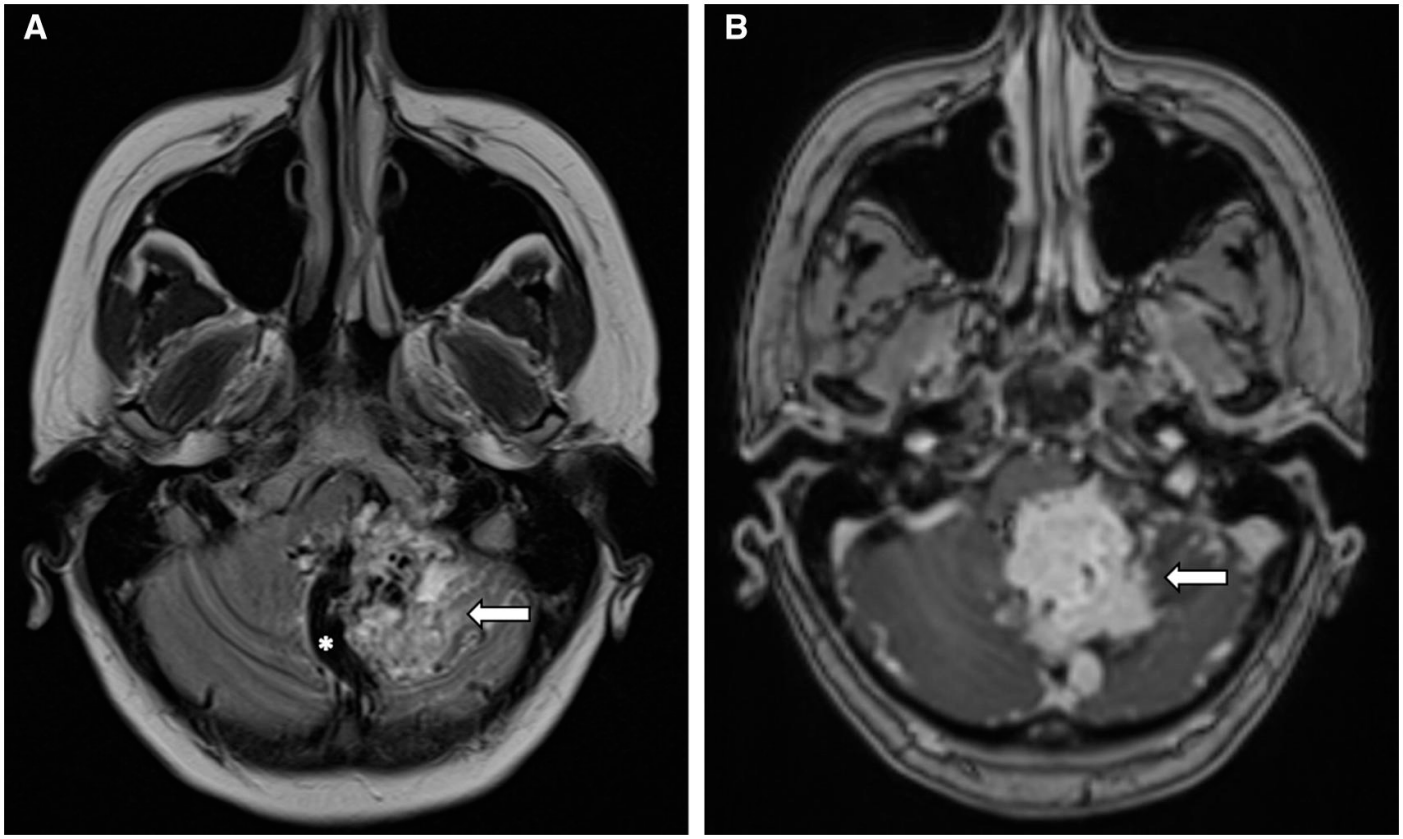
Cerebellar haemangioblastoma. (A) Axial T2-weighted MR image shows hyperintense multilobulated intraaxial mass (arrow) in the left cerebellar hemisphere, and serpiginous flow voids (asterisk). (B) Axial contrast-enhanced T1-weighted MR image shows solid avid enhancement (arrow).

**Figure 3.**
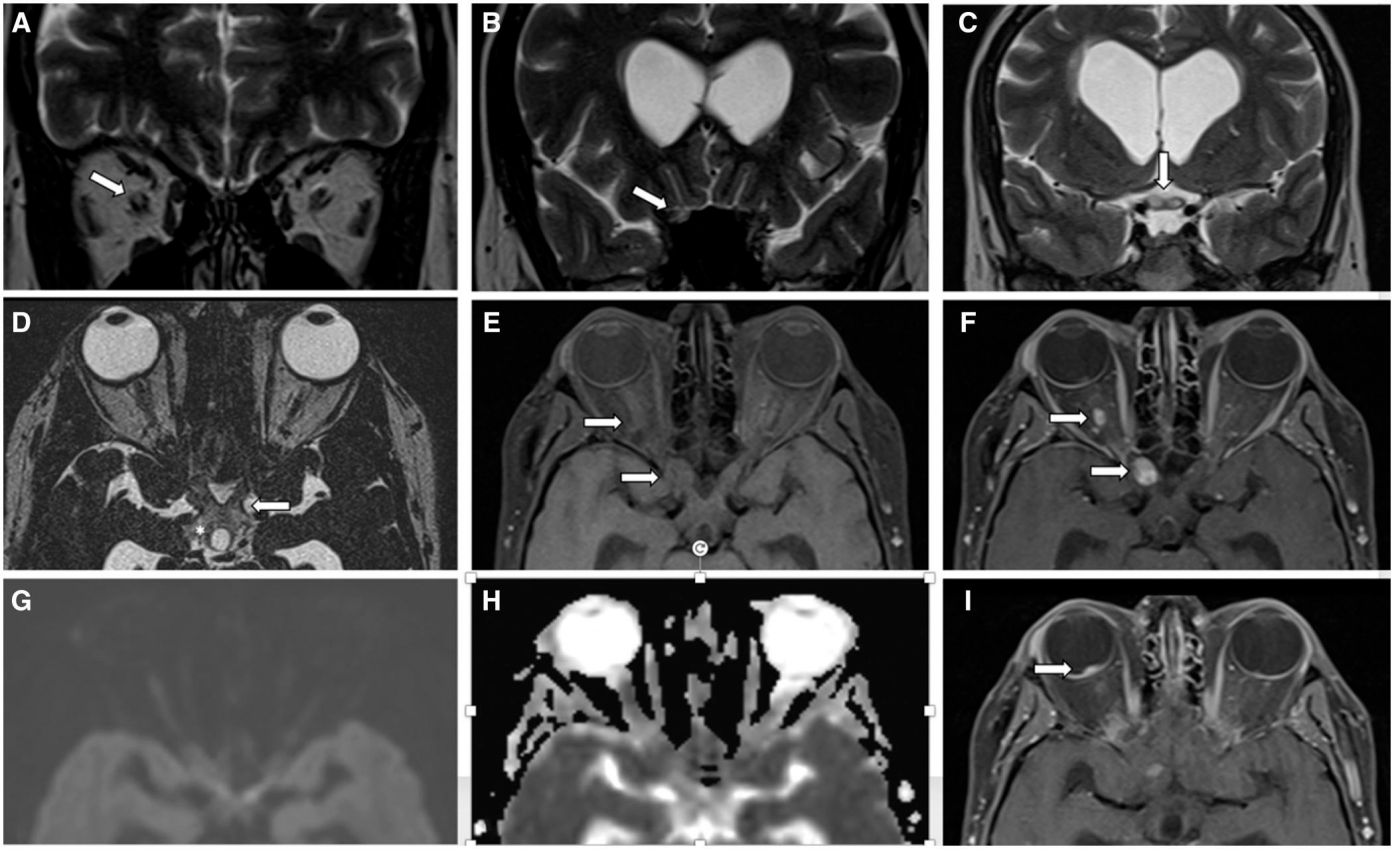
Haemangioblastomas of the optic nerve and retina. Axial (A) and coronal (B) T2-weighted MRI of the orbit show a hyperintense right optic nerve in intraorbital and intracanalicular segment (arrows), and coronal (C) and axial (D) T2-weighted MRI show hyperintense optic chiasm (arrow) with extension to optic radiation bilaterally (asterisk). Axial T1-weighted MR image (E) and contrast-enhanced T1-weighted MR image (F) show focal lesions with avid enhancement in the intraorbital and intracanalicular segment of the right optic nerve. No restriction in DWI and ADC map (F, G). Axial (E) contrast-enhanced T1-weighted MR image shows an enhancing nodular focal lesion on the optic disc (arrow).

**Figure 4.**
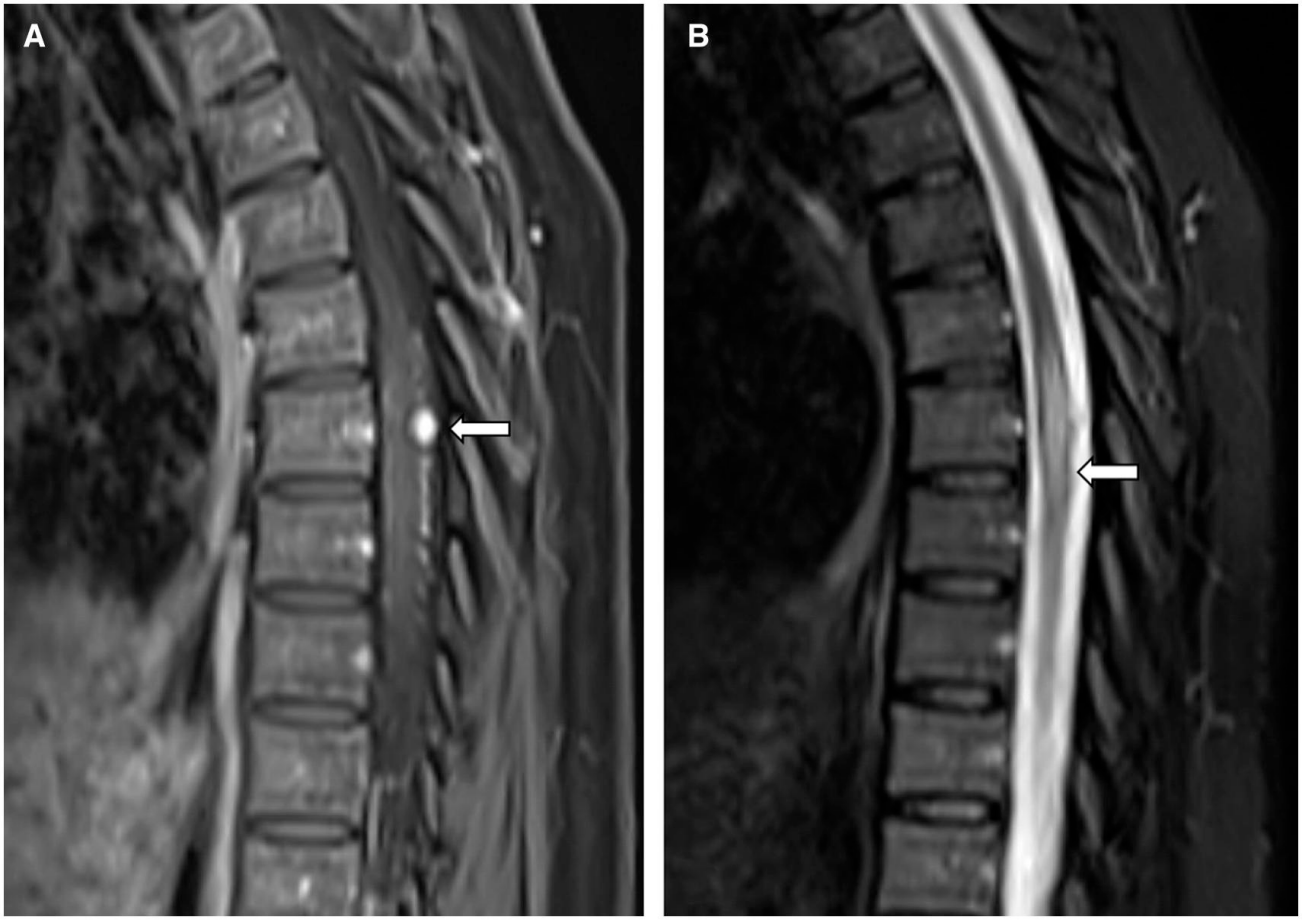
Medular haemangioblastoma. (A) Axial contrast-enhanced T1-weighted MR image of the dorsal spine shows intramedular focal lesions with avid enhancement in T7 (arrow). (B) Axial T2-weighted MR image shows perilesional spinal cord edema (arrow).

Subsequently, a contrast-enhanced brain MRI (Figure 2) with emphasis on orbits (Figure 3) and spine MRI (Figure 4) were made, confirming an intraaxial mass in the left cerebellar hemisphere associated with secondary chronic obstructive hydrocephalus, focal lesions in the optic disc and in the right retrobulbar optic nerve, associated with edema that extend to the chiasm, optic tracts, and dorsal intramedullary lesion at T7, respectively, suggestive of haemangioblastomas.

The patient was evaluated by ophthalmology where a raised vascularized lesion with fibrous tissue on the surface was found in the fundus, associated with central pigmentary changes. In addition, there was an inclination of the nerve and nasal exudation to the disc, diagnosing retinal haemangiomas. No other lesions in the periphery or macula were observed. Due to the family history of the father, the personal history, and the findings on physical examination and imaging, a VHL syndrome was considered with tumour involvement due to retina, optic nerve, and posterior fossa haemangioblastomas. In the neurosurgery staff, it was considered unresectable, so it was decided to manage the hydrocephalus with a Hakim valve and to provide radiosurgery as a treatment option for ophthalmic involvement.

## Discussion

VHL disease is an autosomal dominant hereditary multisystem syndrome, which owes its name to the German ophthalmologist Eugen von Hippel who in 1904 described this rare retinal disorder and named it “angiomatosis of the retina”. Years later, the Swedish pathologist and bacteriologist Arvid Vilhelm Lindau described the association between angiomatosis of the retina and haemangioblastomas of the cerebellum and other parts of the central nervous system and called it “Angiomatosis of the central nervous system”.^2^ VHL disease is extremely penetrant, and although it is hereditary in most cases, new mutations are the cause of up to 20% of cases.

Manifestations include haemangioblastoma of the CNS and spinal cord, renal cell cancer (RCC), RH, pheochromocytoma, epididymal and broad ligament cystadenomas, endolymphatic sac tumour, pancreatic neuroendocrine tumours, and renal and pancreatic cysts. The approximate incidence of VHL disease is 1 in 36 000 live births and penetration is greater than 90% at 65 years of age. Most deaths are caused by metastatic RCC and CNS lesions, and even though clinical treatments have improved, the Life expectancy with VHL remains low between 40 and 52 years. Haemangioblastomas in the CNS are usually found in the cerebellum (50%-60%), spinal cord (40%-50%), and brain stem (10%-20%), but they have also been found in the pituitary stalk (2%-4%). Supratentorial haemangioblastomas are uncommon (<2%-5%) and those of the optic nerve are extremely rare.^4^^,^^5^

Optic nerve haemangioblastoma was first described by Verga in 1930 based on the histopathological findings of a necropsy specimen showing tiny capillaries and larger vessels lined by endothelium and supported by a reticulin structure, with most of the tumour located in the optical nerve without involving the leptomeninges and without a clear tumour capsule.^2^^,^^6^

VHL disease has been associated with some ocular conditions. RH is a benign vascular neoplastic lesion that originates in the neurosensory retina or optic disc, being one of the most frequent and earliest manifestations, approximately in 68% of patients with VHL, increasing with age, with a probability of presenting in 80% of patients older than 80 years.^4^^,^^7^ However, ONH is an extremely rare tumour, with only 40 cases reported since 1940. Although they may have an isolated presentation, up to 70% are associated with VHL syndrome.^2^^,^^4^ Patients usually consult due to loss of vision and exophthalmos, however, cases of asymptomatic patients have also been reported.^1^^,^^6^ Surgical intervention is the primary treatment approach in both conditions and is considered the preferred modality. The efficacy of stereotactic radiosurgery in achieving favourable tumour control over a 5-year period has been demonstrated in these individuals, suggesting it may serve as a viable option for those with significant medical comorbidities or tumours that cannot be surgically removed.^3^

Although contrast-enhanced MRI is considered an optimal diagnostic tool, ONH is still underdiagnosed or undiagnosed, given its low prevalence and similar patterns with other pathologies such as optic nerve glioma (ONG)or optic nerve sheath meningioma (ONSM).^2^ With the advancement of MRI, several sequences have been included for the characterization of these lesions, such as diffusion images and their respective ADC map, MR angiography, and perfusion, which provide new knowledge about the manifestations of image. HON is characterized by the presence of solid, well-defined mass, predominantly hypointense on T1-weighted sequences, hyperintense on T2-weighted images, with postcontrast enhancement and increased signal intensity on apparent attenuation coefficient (ADC) sequences. Structural features including flow voids (highly specific, but not very sensitive) and extensive peritumoural oedema are also widely accepted as suggestive evidence for this pathology.^1^ Retrobulbar haemangioblastomas are often supplied by branches of the ophthalmic artery, the high blood volume and permeability of these tumours result in a disproportionately large amount of oedema for the size of the lesion. This oedema tends to spread along low-resistance white matter pathways and may be evident on T2-weighted sequences and extend from the optic nerve through the chiasm to bilateral optic radiation.^3^ These tumours are highly vascularized and in a relatively confined area such as the optic nerve, the optic nerve sheath appears to serve as a barrier to the outflow of tissue fluid from passive congestion into the microcirculation that leaks into the extracellular space. This fluid, in turn, travels backward along the optic nerve fibre tracts and can extend to the geniculate bodies and beyond. In our patient, the oedema extended to the chiasm and partially to the optic tracts bilaterally, finding few cases in the literature with such extensive oedema.^8^ The dominant solid form and extensive oedema are characteristic features of haemangioblastoma of the optic nerve, distinguishing it from cerebellar haemangioblastoma.^2^

Previous studies have reported elevated levels of rCBV (relative cerebral blood volume) in perfusion sequences specifically in cases of cerebellar haemangioblastoma. However, there is currently a lack of definitive information regarding the association between rCBV and ocular pathology. All this conglomerate of findings would help to distinguish ONH from ONG or ONSM.^1^

Given the improved resolution of MRI, the necessity of cerebral angiography for diagnosing ONH is generally obviated. Nevertheless, previous studies have revealed the presence of a tumour with a significant vascular network that receives its blood supply from various branches of the ophthalmic artery. The late arterial phase demonstrates early venous drainage into the basal vein of Rosenthal. This modality has the potential to be beneficial in the exclusion of optic nerve glioma, as it typically exhibits hypovascularity on angiography without the presence of early draining veins.^3^

An optic pathway glioma (OPG) is classified as a low-grade glioma characterized by the nervés thickened and spindle-shaped appearance and its exhibits a MRI pattern resembling ONH. However, its enhancement is often heterogeneous if there is a cystic component or it does not enhance if there is a solid component. There are three distinct characteristics that aid in distinguishing between them: flow void is more commonly present in the ONH, the presence of peritumoural oedema suggests the possibility of ONH involvement, and the absence of enhancement in the solid component is most often observed in OPG.

Most ONSM are located within the orbit (90%) and exhibit a spherical or tubular thickening of the optic nerve. These tumours appear hyperintense in the T2-weighted images, but display a distinctive sign known as the “tram track” after the administration of contrast medium. This sign is characterized by enhancement along the sheath, while the central portion of the optic nerve appears hypointense. Calcifications are detectable in head CT.^2^

Less frequently observed neoplasms such as gangliogliomas, medulloepitheliomas, and vascular tumours have been found to be linked to the optic pathway exhibiting comparable characteristics to those of OPGs.^9^

Leptomeningeal dissemination of haemangioblastomas is extremely rare and all occur months or years after the initial resection of the primary tumour, but their location and multiplicity are associated with considerable mortality and neurological morbidity.^5^^,^^10^

The primary therapeutic approach for ONH is surgical resection, which is recommended as the initial treatment option. Stereotactic radiosurgery has been identified as a viable alternative therapeutic approach, particularly in cases where tumours remain incompletely resected, residual, multiple, recurrent, or surgically inaccessible due to unfavourable anatomical characteristics or the presence of medical comorbidities that render invasive procedures excessively hazardous. Stereotactic radiosurgery is a viable approach for the treatment of small ONH and presents the benefit of reduced morbidity in comparison to surgical resection.^11^

In their study, Alvarez et al^11^ analysed the natural progression of retrobulbar haemangioblastomas in a substantial cohort consisting of 250 patients diagnosed with VHL syndrome. The primary objective of their investigation was to assess various aspects related to the manifestation, clinical course, progression, MRI characteristics, treatment modalities, and subsequent response of retrobulbar haemangioblastomas. From this cohort, the researchers specifically selected 18 patients with retrobulbar haemangioblastomas, among whom 10 exhibited associated symptoms. Notably, surgical intervention resulted in improvement for 5 of these patients. In asymptomatic patients, ONH remained radiographically stable for a long period of time.^11^

In our case report, the patient’s familial history (specifically, the father's likely diagnosis of VHL personal medical history of pheochromocytomas, associated with the presence of cerebellar haemangioblastoma, RH, and the observed optic nerve findings, proved to be highly definitive and significant in establishing the diagnosis. Patients who have a personal or family history of VHL syndrome, visual disturbances and radiological evidence of optic nerve lesions should be considered and ONH are highly probable.^12^ The patient underwent radiosurgery management for her ocular pathology and a ventricular shunt was performed to manage her obstructive hydrocephalus.

## Learning points

- Optic nerve haemangioblastoma is entity is extremely rare, with only 40 cases reported since 1940, and up to 70% are associated with VHL syndrome.
- Conglomerate of MRI findings would help to distinguish this type of lesions from meningioma, glioma, and other neurological tumours.
- Surgical management is the mainstay of therapy and is the modality of choice.
- Stereotactic radiosurgery can offer satisfactory 5-year tumour control in these patients with severe medical comorbidities or surgically unresectable tumours.
